# Impact of *Aedes aegypti* V1016I and F1534C knockdown resistance genotypes on operational interventions

**DOI:** 10.1038/s41598-025-94738-z

**Published:** 2025-03-24

**Authors:** Alden S. Estep, Neil D. Sanscrainte, Muhammad Farooq, Keira J. Lucas, Rebecca L. Heinig, Edmund J. Norris, James J. Becnel

**Affiliations:** 1 USDA-ARS Center for Medical Agricultural and Veterinary Entomology, Mosquito & Fly Research Unit, Gainesville, FL USA; 2 Anastasia Mosquito Control District, St. Augustine, FL USA; 3 Collier Mosquito Control District, Naples, FL USA; 4 Thermacell Repellents, Inc., Hampton, FL USA

**Keywords:** *Aedes aegypti*, Insecticide resistance, Knockdown resistance (*kdr*), Operational efficacy, Voltage-gated sodium channel, Haplotypes, Entomology

## Abstract

Resistance to pyrethroids is common in *Aedes aegypti* populations. Mutations in the voltage gated sodium channel have an influence on the insecticide resistance (IR) phenotype. In much of the Western hemisphere, two of these knockdown resistance (*kdr)* mutations, V1016I and F1534C, result in six *kdr* genotype combinations in field populations. Strong pyrethroid IR and the failure of permethrin treated uniforms have been linked to the presence of the homozygous double *kdr* genotype (IICC) but the effects of the other five *kdr* combinations have not been rigorously examined, particularly with regard to operational efficacy. To better understand the impacts of these common *kdr* genotypes, we isolated three *kdr* haplotypes (VF, VC, & IC) from a field collected strain to produce six *Ae. aegypti* isoline strains with all the common V1016I/F1534C *kdr* genotypes. We then characterized the effects of these genotypes by CDC bottle bioassay and topical application and found increasing resistance to permethrin and deltamethrin as the number of IC haplotypes increased. Neither enzymatic activity nor malathion resistance increased with increasing pyrethroid resistance. We then assessed the operational impacts of these *kdr* genotypes. Field and wind tunnel spray of a pyrethrin formulation showed that even moderate resistance could significantly reduce knockdown and mortality. Studies with a synergized pyrethroid formulation showed effective recovery of mortality against all genotypes except for the IICC. In human bite studies, one or two IC haplotypes compromised the efficacy of permethrin treated fabrics. This study demonstrates that *kdr* mutations have distinct phenotypic effects in both the laboratory and operational interventions, and that the strength of pyrethroid resistance varies with the number of IC haplotypes present. Assessing *kdr* genotype is therefore critical for understanding IR in *Ae. aegypti*.

## Introduction

Insecticide resistance (IR) to pyrethroids is found worldwide in *Aedes aegypti* (L.). Populations are generally resistant to pyrethroids when laboratory IR testing is conducted, though the field relevance of this resistance is often unclear^1–6^. Because IR can dramatically reduce the efficacy of control operations critical to breaking disease transmission cycles, national and international agencies recommend regular IR testing as an important part of vector management^7–10^.

Enzymatic detoxification and target-site resistance are the two primary IR mechanisms and are the most frequently assessed^1–4^. Numerous enzyme families have demonstrated the ability to degrade model substrates in biochemical assays and in other systems, but there is no clear pattern of overexpression of cytochrome P450s (CYP450), glutathione-S-transferases (GST) and esterase families in resistant field populations of *Ae. aegypti*^4,11–13^. Additionally, several studies have also shown increased enzymatic activity in *Ae. aegypti*, but these assays do not always correlate with increasing resistance levels and, when coupled with relatively low synergist ratio measurements even in highly resistant adult populations, means that global biochemical measurements of elevated activities may be due to processes other than IR^4,9,14–16^. Notably though, while there may not be a particular signature enzymatic response in *Ae. aegypti* to an insecticide, there is always some enzymatic activity that contributes to the IR phenotype, even in laboratory strains^4,12,15^.

Target-site resistance to pyrethroids from individual knockdown resistance *(kdr)* mutations, or from an ensemble of *kdr* mutations, appears to be an important contributor to the overall level of IR in *Aedes aegypti*. Many voltage-gated sodium channel (*VGSC)* single nucleotide polymorphisms (SNPs) have been described; combinations of S989P, V1016G and F1534C mutations are primarily found in the Indo-Pacific while in the Western hemisphere, combinations of V410L, V1016I and F1534C have been widely described^2,4^. These groups of SNPs, with the exception of F1534C, have long remained geographically isolated, although recent studies have detected some breakdown of this separation^17–19^. Studies indicate that the intensity of IR depends on the specific complement of *kdr* SNPs present; homozygous combinations of 989P/1016G and 1016I/1534C (genotype: IICC) appear to result in strong resistance in the Eastern and Western hemispheres respectively^2,4,20^.

A strong correlation between resistance ratio (RR) and population IICC frequency has been shown in US field populations^21,22^. Additionally, the longest surviving California *Ae. aegypti* in bottle bioassays had the IICC genotype^23^. The same study also showed 1534C was nearly fixed and that several other mutations were present (such as V410L, T915K and S723T), but only the IICC combination partitioned solely with the mosquitoes with the longest knockdown times^23^. Multi-year *kdr* monitoring studies paired with treatment records show that *kdr* frequencies can rapidly increase in response to continuous treatment with pyrethroids and can be slow to drop when pressure is removed^24–27^.

The goal of laboratory IR testing is ultimately to provide information to improve operational control, reduce vector borne disease risk and limit nuisance issues due to excessive mosquito biting^9,28^. However, the links between laboratory detected IR, the genetic markers of IR and field efficacy are not well examined in *Ae. aegypti*, thus the usefulness of laboratory derived IR information is limited^29–31^. To better characterize these links between lab IR testing, resistance mechanisms and field performance, we generated *kdr* isoline strains from a moderately resistant field collected *Ae. aegypti* strain to partition, characterize and quantify the toxicologic and operational response of six commonly occurring Western hemisphere combinations of the V1016I and F1534C *kdr* SNPs (VVFF, VVFC, VVCC, VIFC, VICC, IICC).

## Results

### Characterization of phenotypic resistance

**Fig. 1:**
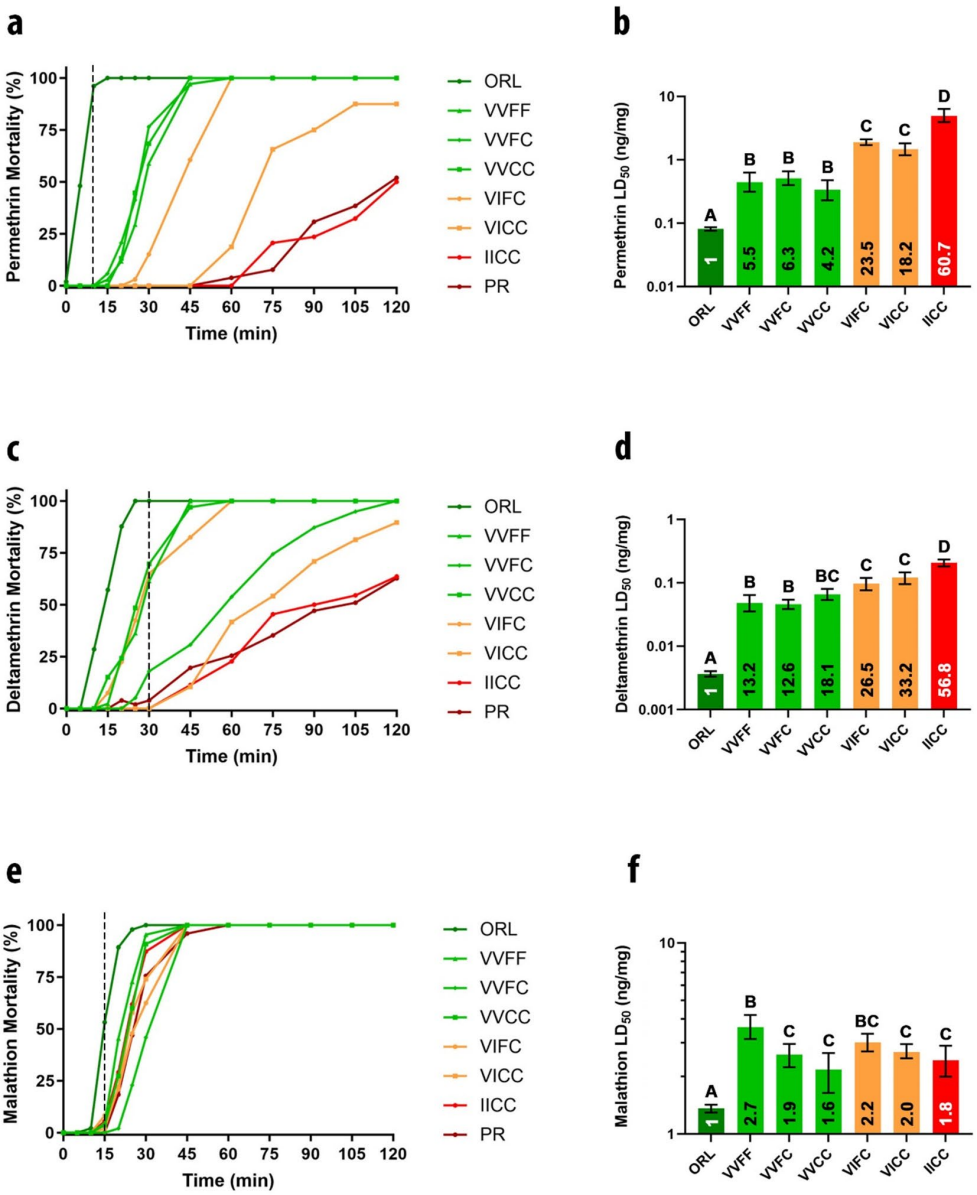
Laboratory assessment and quantification of insecticide resistance phenotype in *Aedes aegypti* strains with distinct knockdown resistance (*kdr*) genotypes. Strains were assessed for insecticide resistance using standard CDC bottle bioassay (panels **a**, **c**, **e**) and the resistance levels were quantified by topical application (panels **b**, **d**, **f**). Standard CDC active ingredient diagnostic times for *Ae. aegypti* are noted with a dashed black line (panels **a**, **c**, **e**) and resistance ratios (calculated by dividing the LD_50_ by the LD_50_ of the ORL susceptible strain) are noted on each column (panels **b**, **d**, **f**). (**a**) CDC bottle bioassay testing of permethrin at 43 µg/bottle. (**b**) Topical application of permethrin. (**c**) CDC bottle bioassay testing of deltamethrin at 0.75 µg/bottle. (**d**) Topical application of deltamethrin. (**e**) CDC bottle bioassay testing of malathion at 400 µg/bottle. (**f**) Topical application of malathion. Error bars on median lethal doses represent 95% confidence intervals and colors denote genotypes with either 0 (green), 1 (orange), or 2 (red) copies of the 1016I and 1534 C *kdr* haplotype. Differing letters in panels **b**, **d**, **f** indicate significant differences.

To assess the presence and intensity of phenotypic resistance, we conducted CDC bottle bioassay testing using females from each *kdr* genotype-specific strain with technical grade permethrin, deltamethrin and malathion. All strains, except the laboratory susceptible Orlando1952 (ORL) strain, had mortality less than 90% at the diagnostic time (DT) of 10 min (the CDC threshold for resistance) to the type I pyrethroid permethrin (Fig. 1A). Over the course of the two-hour assay period, the curves segregated. Strains with no copies of the IC haplotype (VVFF, VVFC and VVCC) reached complete mortality within 45 min. Strain VIFC reached complete mortality within 60 min while VICC reached only 90% during the two-hour period. The IICC strain and laboratory resistant Puerto Rico (PR) strain only reached 50% and 51.2% mortality during the two-hour exposure, respectively. Topical application to quantify IR intensity and produce RRs showed a similar trend (Fig. 1B). The ORL strain had the lowest LD_50_ at 0.081 (0.076–0.086) ng/mg, matching previous LD_50_ determinations for this strain^15,21,22^. The three isoline strains with no IC haplotype had LD_50_s different from that of the ORL strain but of low resistance (RR = 4–6) with overlapping 95% confidence intervals (CI) indicating they were not different from one another. Strains containing one IC haplotype (VIFC and VICC) had LD_50_s about 20-fold higher (RR = 18–23) than the ORL strain. The LD_50_ of the IICC strain, which contains two copies of the IC haplotype, had an LD_50_ of 4.92 ng/mg (RR ~ 60) and was commensurate with that previously measured for the PR strain LD_50_^15^.

Similar testing of the type II pyrethroid deltamethrin at the standard DD of 0.75 µg/bottle demonstrated a similar pattern. The ORL strain was completely killed while all others were considered resistant at the 30 min DT (Fig. 1C). We observed 60–70% mortality in the VVFF, VVCC and VIFC strains at the deltamethrin DT but little mortality in the other strains. Two hour mortality followed the same pattern as permethrin with complete mortality in four strains, 85% mortality in the VICC strain and about 60% mortality in the IICC and PR strains. Similar to permethrin, we observed a stratification in the time to reach maximum mortality that aligned with the number of IC haplotypes present. Topical application with deltamethrin showed the isolines to have median LD_50_s higher than the ORL lab strain with similar groupings by number of IC haplotype copies, as was observed with permethrin (Fig. 1D). The VVFF, VVFC and VVCC strains had overlapping CIs that ranged from 0.35 ng/mg to 0.80 ng/mg resulting in RRs of 12–18 compared to the ORL strain. Distinct from these were the single-IC haplotype containing strains (VIFC and VICC), with 26.5-33-fold resistance (not significantly different from each other). As with permethrin, the dilocus mutant homozygote, IICC, was > 56-fold resistant and distinct from all other strains.

Bottle bioassay testing with the organophosphate (OP) malathion showed all strains, including the laboratory susceptible strain, were resistant at the DT of 15 min (Fig. 1E). However, by 45 min, all strains were dead except the PR strain which showed > 90% mortality. We also determined LD_50_s for malathion because it is widely used as an adulticide for operational mosquito control^32,33^. Topical bioassay testing showed that the *kdr* isolines were resistant compared to the ORL but at very low intensity with RR < 3 for each strain and they were not distinct from one another (Fig. 1F). This indicates that malathion resistance is minimal in these *kdr* isoline strains.

### Characterization of resistance mechanisms

**Fig. 2:**
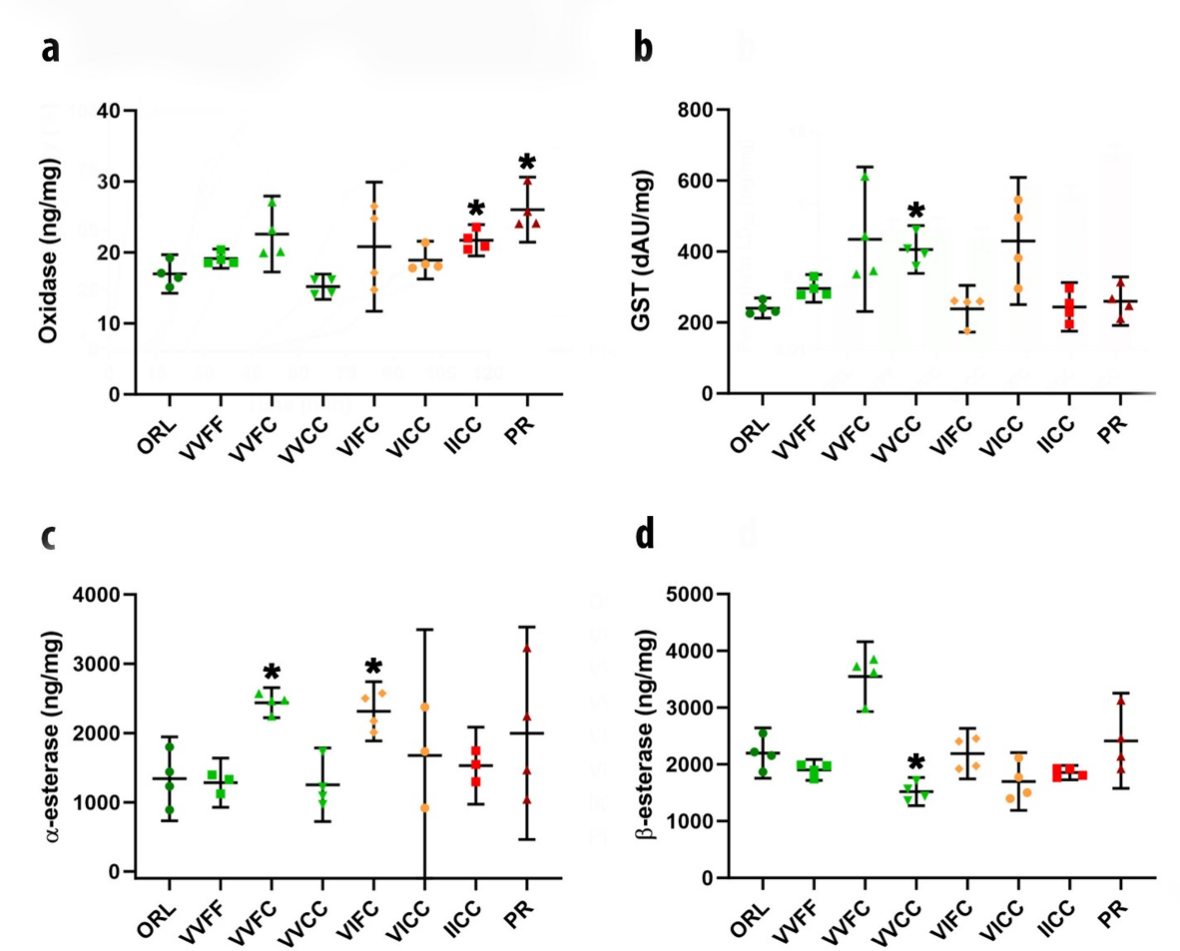
Biochemical assessment of enzymatic activity in *Aedes aegypti* strains with distinct knockdown resistance (*kdr*) genotypes. Strains were assessed for enzymatic activity (panels a–d) using biochemical assays. (**a**) Oxidase activity. (**b**) Glutathione-S-transferase activity. (**c**) α-esterase activity. (**d**) β-esterase activity. Error bars on biochemical assays (panels **a**–**d**) represent 95% confidence intervals (CIs) and colors denote genotypes with either 0 (green), 1 (orange), or 2 (red) copies of the 1016I and 1534C *kdr* haplotype. Non overlap of 95% CIs is considered significant. Significant differences relative to the control ORL strain are marked by asterisks.

Phenotypic testing indicated varied IR intensity to pyrethroids but not to the representative OP malathion. To better understand the resistance mechanisms and determine relative contributions from *kdr* versus enzymatic activity, we conducted biochemical assays to measure potential detoxifying enzyme activity for the *kdr* isolines and control strains. These showed no pattern of increasing enzymatic activity as phenotypic resistance increased in the isoline strains (Figs. 2A–D). Enzymatic activity also was not increased in the PR strain relative to the *kdr* isolines. However, there were sporadic findings of small significant increases. Assays for oxidase activity did indicate significantly increased activity (Brown-Forsythe ANOVA; F = 5.968 (7.000, 8.922), *p* < 0.0083) in the IICC (*p* = 0.027) and PR strains (*p* = 0.015) when compared to the laboratory ORL strain (Fig. 2A). With respect to GST activity, only the VVCC strain had significantly increased activity (*p* = 0.0096) relative to the ORL strain (BF ANOVA F = 6.793 (7.000, 9.364), *p* = 0.0046) (Fig. 2B). For α-esterase activity, we observed minimally significant increases in activity (BF ANOVA F = 3.593 (7.000, 7.954), *p* = 0.0472) in the VVFC (*p* = 0.0263) and VIFC (*p* = 0.0437) isolines (Fig. 2C). Beta-esterase activity was significantly increased (BF ANOVA F = 17.41 (7.000, 12.99), *p* < 0.0001) in the VVFC isoline (*p* = 0.0126) and decreased in the VVCC isoline (*p* = 0.0413) (Fig. 2D).

**Fig. 3:**
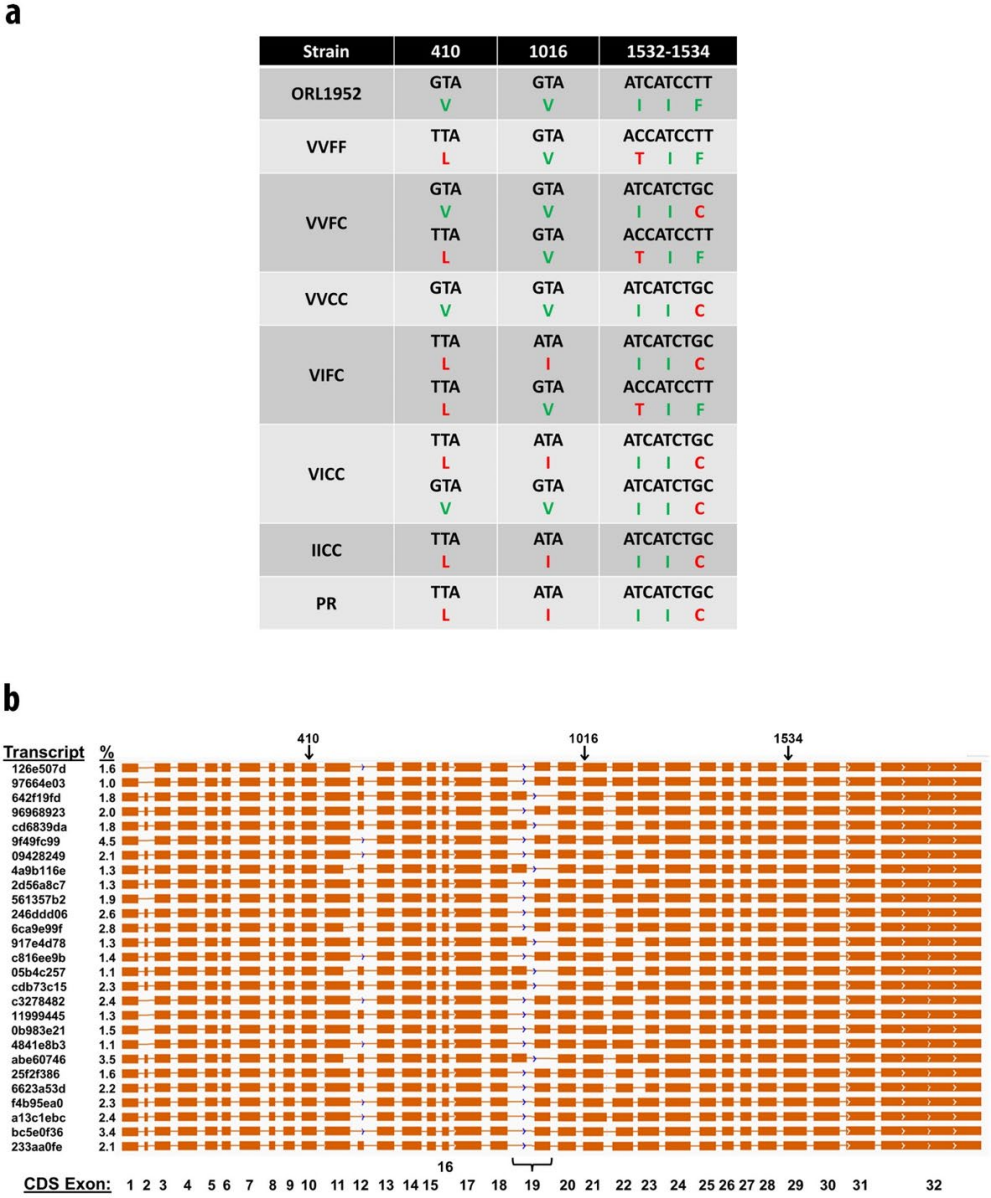
Knockdown resistance (*kdr*) genotype of *Aedes aegypti* strains in this study. (**a**) Verification of expressed *kdr* genotype and translation for each strain at positions 410, 1016, and 1532–1534 are shown with the heterozygous genotypes made up of two distinct haplotypes. Wildtype (no *kdr*) translations are shown in green, mutants in red. (**b**) Schematic of coding sequence (CDS) exon usage and location of *kdr* SNPs considered in this study. Data for this analysis is found in the NCBI Sequence Read Archive under BioProject ID PRJNA832328.

Nanopore sequencing of the > 6 kb coding region of the *VGSC* was conducted after amplification from the six *kdr* isoline and two control strains (ORL and PR). The resulting sequences from the six *kdr* isolines confirmed the expected genotypes were reflected in the expressed RNA (Fig. 3A). We observed 1016V paired with 1534F (VF), 1016V paired with 1534C (VC), and 1016I paired with 1534C (IC). We did not find evidence of the rare allele combination of 1016I with 1534F (IF)^21,24,27^. Strain specific results showed that the expected alleles were present in all the strains and clearly detectable in the heterozygous isolines VVFC, VIFC and VICC (Fig. 3A). We further observed that the recently described 410L mutation was present in both our PR control strain (which was collected in San Juan, Puerto Rico in 2012) and all the *kdr* isolines except the VVCC strain. Examining the individual reads showed the 410L SNP was present on both the VF haplotype and the IC haplotype, but we did not detect it present with the VC allele in these strains. While we observed numerous other synonymous SNPs, we also observed a novel 1532 I to T SNP (ATC◊ACC) in the VF haplotype. This 1532T was not present in the VC or IC haplotype and was not present in the ORL or PR laboratory strains.

Sequencing reads that encompassed the full-length of the *VGSC* coding region enabled unambiguous assignment of the relationship between the SNPs along individual transcripts as well as allowing more insight into the *VGSC* coding population than would be possible by a cloning and Sanger sequencing or a traditional next generation sequencing strategy. As observed during sequencing of the genome and transcriptome of the Liverpool strain used to produce the L5 genome reference (Accession: GCF_002204515.2), each strain here had numerous alternate transcripts with varied exon usage across the coding sequence (NCBI Accessions: SRX15010228- SRX15010235) (Fig. 3B). We observed that the strains were highly similar (> 99%) to the complete nucleotide sequence of the canonical L5 *VGSC* and that patterns of SNPs moved with certain alleles. An area that appeared to contain many SNPs was between positions 2700–2900 (exon 19) of the alignment and was observed in all strains. However, subsequent alignment against the reference genome showed this region is not due to nucleotide polymorphism but is instead due to alternate exon usage of two mutually exclusive exons as described previously in *Drosophila*^34,35^. The combination of SNPs in this region are primarily silent so the importance of these mutually exclusive exons is unclear and is an area for further study.

Examination of the three *kdr* haplotypes (VF, VC, IC) showed both common and distinct features. The VVFF and VVFC strains contained the VF haplotype which allowed comparison of the same haplotype in the very susceptible ORL laboratory strain. Notably, the VF haplotype in the *kdr* strains had numerous SNPs when compared to the VF allele of the ORL strain, including the 410L and the 1532T SNPs. Though variable from one another, both had numerous SNPs when compared to the transcript model as well. The VC haplotype was more similar to the laboratory ORL strain VF haplotype though not identical. The V410L SNP was not found in reads with the VC haplotype. The IC sequence data from the isolines and from the PR strain were very similar with one SNP at about 5600 in the PR strain. While most exons were present in every sample, exon 2 (bases 148–181), exon 11 (bases 1591–1611) and exon 12 (bases 1612–1674) were found in various combinations (Fig. 3B). As noted above, exon 19 appears to be derived from mutually exclusive but distinct colinear mappings in the genome assembly. Notably, the exons containing the site of the 410, 1016 and 1534 SNPs were observed in all the various transcripts (Fig. 3B).

### Effects of Kdr genotype on operational adulticide formulations

**Fig. 4:**
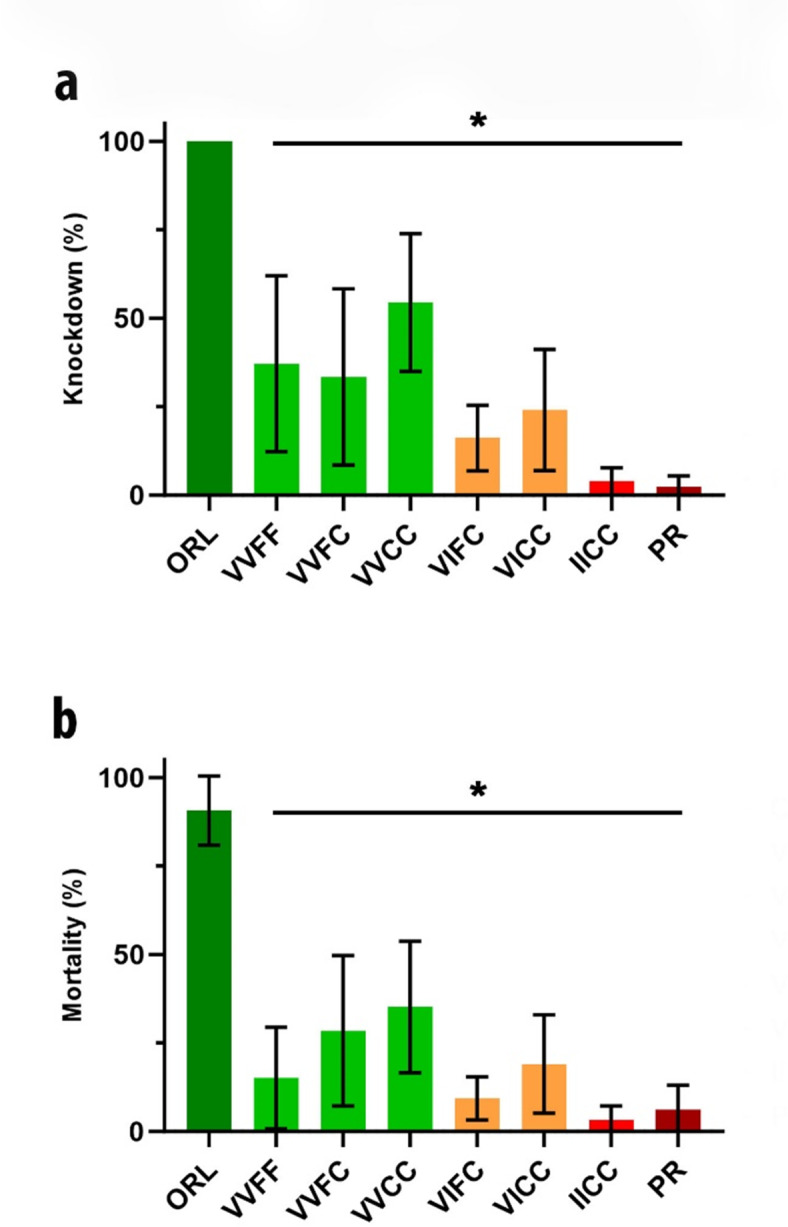
Efficacy of commercial pyrethrin formulation by aerial spray. Strains were assessed for (**a**) one hour knockdown and (**b**) 24 h mortality after application of commercial formulations by MD500 helicopter. Error bars represent standard error from nine field cages of each strain and colors denote genotypes with either 0 (green), 1 (orange), or 2 (red) copies of the 1016I and 1534C knockdown resistance haplotype. Significant differences relative to the control ORL strain are marked by asterisks. Columns lacking error bars had standard error of 0.

We assessed the performance of these strains under normal operational conditions by conducting a semi-field trial using caged mosquitoes in collaboration with Collier Mosquito Control District. An unsynergized formulation, containing pyrethrins, was applied using an MD-500 helicopter equipped with a single electric atomizer at the application rate of 0.88 oz/acre (0.0025 lb pyrethrins/acre), according to label specifications. Cages for each strain were exposed to the same application event. At one hour post-application, we observed 100% knockdown in the nine cages containing the ORL strain, indicating good coverage of the entire application area. All the isoline strains and the PR strain showed significantly less knockdown (F (3.229, 25.83) = 26.82, *P* < 0.0001) than the ORL strain (Fig. 4A). Strain VVCC showed the highest knockdown among the isoline strains at 54%. Notably, the IICC and PR strains had less than 10% knockdown. Mortality at 24 h showed some recovery, but all strains had significantly less mortality than observed against the ORL strain (Fig. 4B).

**Fig. 5:**
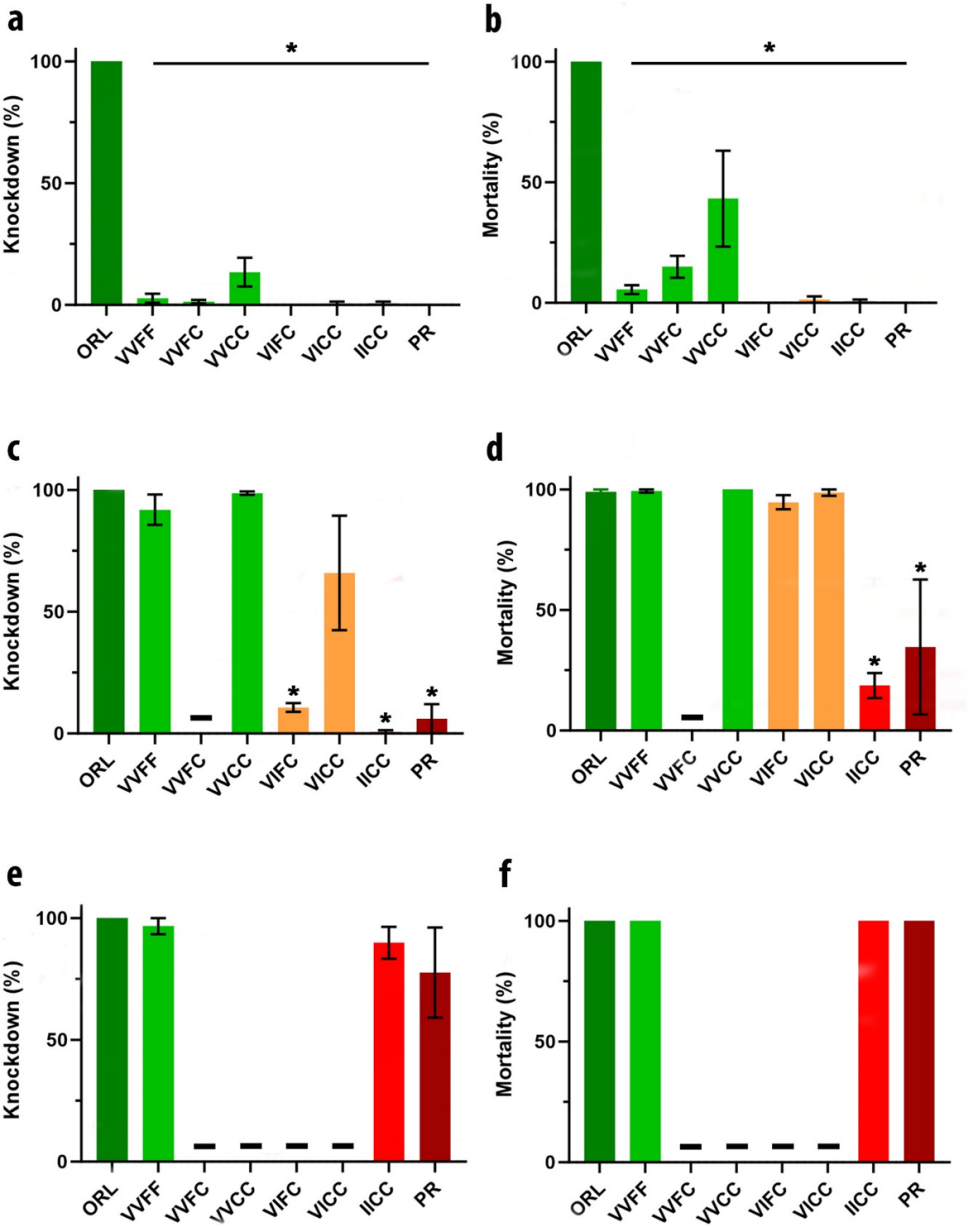
Efficacy of commercial mosquito control formulations against *Aedes aegypti* strains with distinct knockdown resistance (*kdr*) genotypes in a wind tunnel. Strains were assessed for one hour knockdown (panels **a**, **c**, **e**) and 24 h mortality (panels **b**, **d**, **f**) after exposure to commercial formulations at label rate doses. (**a**) One hour knockdown after exposure to a natural pyrethrin formulation at 0.88 oz/acre. (**b**) 24 h mortality after exposure to a natural pyrethrin formulation. (**c**) One hour knockdown after exposure to a PBO synergized permethrin formulation at 0.83 oz/acre. (**d**) 24 h mortality after exposure to a PBO synergized permethrin formulation. (**e**) One hour knockdown after exposure to a malathion formulation at 0.88 oz/acre. (**f**) 24 h mortality after exposure to a malathion formulation. Error bars represent standard error of three or four replicates and columns lacking error bars had standard error of 0. Colors denote genotypes with either 0 (green), 1 (orange), or 2 (red) copies of the 1016I and 1534C *kdr* haplotype. Columns with “-“ indicate strains and active ingredient combinations not tested. Significant differences relative to the control ORL strain are marked by asterisks.

We conducted replicated testing with a wider range of active ingredients and formulations by partnering with Anastasia Mosquito Control District to do wind tunnel testing. To directly compare results, we tested the same unsynergized pyrethrin formulation (Fig. 5A and B) in the wind tunnel that was used in the aerial spray. Both methods produced similar results for all eight strains (compare Figs. 4A and B and 5A and B) at a dose equivalent to the spray flux/sq ft as the label rate of 0.88 oz/acre. We saw complete knockdown of the ORL strain at one hour across the four replicate wind tunnel sprays (Fig. 5A). The non-IC strains averaged slightly less knockdown than observed in the aerial spray, but all averaged less than 50%. In the wind tunnel, the strains containing one or two copies of the IC allele averaged less than 10% knockdown. Twenty four hour mortality was also low with 90% or more of the exposed mosquitoes surviving the spray (Fig. 5B).

We also tested a permethrin formulation synergized with PBO in the wind tunnel equivalent to the label rate of 0.51 oz/acre (Fig. 5C). The PBO synergized product did increase knockdown to near 100% in strains lacking the IC haplotype (VVFF and VVCC; VVFC not tested). Knockdown of the single IC haplotypes was more variable, averaging only about 15% in VIFC but about 60% in VICC. This synergized product did not result in appreciable knockdown against the IICC or the PR strain, both of which include two copies of the IC allele. The no-IC strains and the one-IC strains were effectively controlled with nearly 100% mortality by 24 h (Fig. 5D). Efficacy against IICC and PR was only slightly improved, and mortality remained less than 35%.

Based on the topical results, which indicated good efficacy with an OP, we also tested a malathion formulation in the wind tunnel at a rate equivalent to the label rate of 2.1 oz/acre. We tested only four strains (control: ORL and PR; isoline: VVFF and IICC) which are representative of the extremes of pyrethroid resistance. One hour knockdown was high for all four tested strains with the ORL and VVFF at nearly 100% (Fig. 5E). The IICC strain was similar but more variable and the PR strain was also variable but with an average knockdown ~ 75%. All four strains were effectively controlled at 24 h with 100% mortality (Fig. 5F). We observed no differences in response to malathion regardless of the level of pyrethroid resistance.

### Effects of Kdr genotypes on permethrin treated fabric

**Fig. 6:**
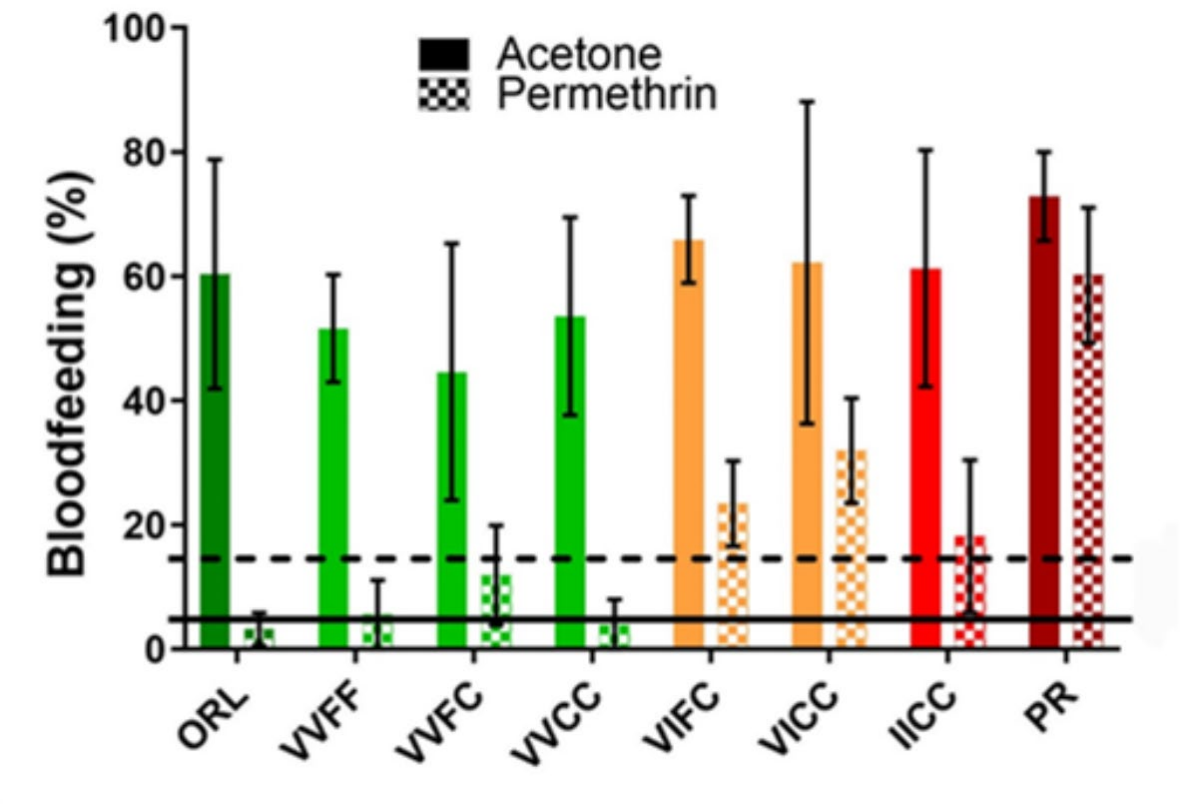
Efficacy of permethrin treated fabric to prevent biting of host-seeking *Aedes aegypti*. Properly consenting volunteers were exposed to knockdown resistance (*kdr*) genotype specific strains while wearing sleeves of standard US military untreated FRACU3 fabric or permethrin treated (0.52% by weight) FRACU3 fabric under an approved human use protocol (IRB#201700023). Blood feeding percentage was assessed after a 15 min exposure period. Error bars represent standard error and colors denote genotypes with either 0 (green), 1 (orange), or 2 (red) copies of the 1016I and 1534 C *kdr* haplotype. The solid and dashed horizontal lines indicate the optimal and minimum efficacy percentage expected by specification MIL-PRF-32659^37^.

Standardized bite protection assays were conducted with these isoline strains on human volunteers using an Institutional Review Board (IRB) approved protocol to assess efficacy. Fire Retardant Army Combat Uniform 3 (FRACU3) material either untreated or treated at the standard dose of permethrin was used. Volunteers placed material covered arms into cages of host-seeking females for 15 min exposures. Bite through of untreated FRACU3 ranged from 45 to 73% for all strains with no observed significant differences between the strains (Fig. 6). Bite through of the permethrin treated FRACU3 fabric varied by strain. The control strains, which have been tested in this assay previously, gave the expected results with significantly reduced biting of the ORL strain (3.2% vs. 60.3%, *p* = 0.036) and no reduction in the PR strain (60.2% vs. 72.8%, *p* = 0.24)^16,36^. The *kdr* isoline strains with no copies of the IC haplotype resulted in less than 15% bite through, which is similar protection to that observed for the ORL strain. The isolines with one or two copies of the IC haplotype each averaged about 20-30% bite through. Variation in this assay was high as various volunteers were relatively more or less attractive to mosquitoes but the presence of even one copy of the IC haplotype resulted in immediate failure of the minimum 85% efficacy required by the specification^37,38^.

## Discussion

Though *kdr* SNPs have been widely assessed in many *Ae. aegypti* populations, the six V1016I and F1534C combinations common to the Western hemisphere have not been isolated and then characterized for laboratory and operational effects. Previous studies examining specific *kdr* genotypes in laboratory strains, limited mating studies, and genetic assessment of *kdr* in field populations have given clues that, in *Ae. aegypti*, the *kdr* genotype is the major contributor to the overall resistance to pyrethroids^20,21,23,24,39–41^. Thus, the primary goals of this study were to characterize the mechanistic contributions underlying the observed resistance, quantify the toxicological effects of each of the six extant *kdr* genotypes produced by combinations of the V1016I and F1534C SNPs and to understand how these individual genotypes contribute to resistance both in the laboratory and in the field. A further goal was to determine what factors detected in the lab would be a useful surrogate to indicate loss of efficacy in the field.

To examine these issues, this study started with a moderately resistant field population from St. Augustine, FL as the founding source for the six *kdr* genotype-specific strains, or isolines^21^. By pairing pupal exuviae genotyping with an existing melt curve assay method, we were able to select pure lines homozygous for the three haplotypes of the V1016I and F1534C SNPs found in the natural field background. Mass mating of genotyped virgin males and genotyped virgin females from these three pure strains produced generations of the three heterozygous *kdr* genotypes detected in the wild (VVFC, VIFC and VICC).

Laboratory testing of these six *kdr* isolines and the two control strains demonstrated general concordance between CDC bottle bioassay testing and topical application. Although the CDC bottle bioassay is less precise and subject to variation due to uncontrolled factors (i.e. mass, sex and nutritional status), it is more easily accessible for control programs than topical application and is therefore in wide use^4,27,30,42^. Both methods identified resistance to pyrethroids and weak resistance to the organophosphate malathion. The CDC assay was very sensitive to the presence of even low-level resistance. The ability to discriminate resistance intensity in the CDC bottle bioassay at the DD and DT was limited, but when assessing assay mortality at two hours clear differences become evident and are overall in agreement with the topical bioassay. Variation in the bottle bioassay has been observed previously and recent studies indicate that survival analysis may be a statistically valid method to evaluate bottle bioassay data that can account for the individual genotypes and other factors^23,43–45^.

For topical application of permethrin, we observed phenotypic segregation based on the number of IC alleles present; the no-IC haplotype group (VVFF, VVFC and VVCC) all showed low resistance (RR ~ 5 or less), the presence of one copy of the IC haplotype provided about 20-fold resistance and two copies resulted in approximately 60-fold resistance. These results agree with recent studies (i.e. one evaluated the VC and IC haplotypes backcrossed into laboratory strains which resulted in differing resistance intensity) and with previous work with the PR strain (also IICC) that showed ≥ 50-fold resistance^15,21,41^. Topical application of deltamethrin also showed a similar striation based on the number of IC haplotypes present but with more overlap, and the LD_50_ calculated here was similar to that recently published from the same IICC strain^46^. The results of this current study indicate few differences observed in the enzyme assays and the lack of significant differences in malathion LD_50_ in the *kdr* strains both indicate that enzymatic mechanisms likely play a small role in the overall IR and that *kdr* genotype is determinative.

It was also notable that the IICC and PR strains, which both have two copies of the IC haplotype, performed almost the same in the CDC assay and had a similar RR in topical applications^15^. Previous work that quantified permethrin RR in field strains showed similar results, suggesting that the IICC genotype percentage may be a useful predictor of resistance intensity^21,47^. Both assays also showed a clear difference between strains with no copies of the IC haplotype – the VVFF isoline and the ORL. The reasons for this difference between the VVFF and ORL are unknown but may be representative of more than 70 years of colonization for the ORL strain and/or the demonstrated differences due to SNPs outside the 1016 and 1534 locations. Regardless of the reason, caution is required when using legacy lab strains as surrogates for even low resistance field strains as they can vary greatly due to differing genetic backgrounds.

The differences in IR observed in the laboratory persisted when subjecting the *kdr* isolines to the more operationally relevant testing. The unsynergized permethrin formulation was uniformly ineffective against all but the ORL laboratory strain in both aerial and wind tunnel sprays, which indicates that the relatively weak IR from non-*kdr* mechanisms of the no-IC haplotype group was sufficiently above the threshold needed for reduced field efficacy. This further highlights the distinction made above regarding the differing genetic backgrounds between lab strains held in colony for decades and freshly colonized field strains. As in the laboratory, the no-IC haplotype group had more knockdown and mortality than the strains containing one- or two-IC haplotypes. The control ORL strain was completely knocked down and did not recover, indicating that sufficient spray flux made it through the cages and that the differences were not due to poor spray coverage but instead due to differences in the organisms which we attribute to *kdr* genotype.

With the synergized permethrin formulation, the results were more nuanced. The presence of the synergist did have the expected increase in knockdown against the *kdr* isoline strains. Twenty four hour efficacy against the no-IC and the one-IC strains was increased but remained poor against the two-IC strains. While it is clearly beneficial in the short term to include a synergist to increase efficacy, the selective pressure of removing the other five *kdr* genotypes and leaving primarily IICC would have rapid negative effects on population level resistance as the IICC genotype percentage increases in the population. Testing with the synergized formulation is a demonstration of the window of selection concept where the pressure on a population varies within specific portions of the population, in this case by *kdr* genotype^48,49^. This strong selection for the survival of the IICC genotype could lead to a rapid loss of control, particularly where mosquito generations in subtropical and tropical locations can emerge weekly. In contrast, our results demonstrated that a malathion formulation was effective against all *kdr* genotypes and is, for populations like these, a viable option for *Ae. aegypti* operational control when pyrethroid IR from *kdr* is present and an adulticide is needed. It should be noted that malathion resistance has been reported in some populations of *Ae. aegypti*, tested primarily by CDC bottle bioassay, so operational testing should be conducted to ensure OP efficacy^50,51^.

Permethrin-treated fabrics are an important field intervention in the consumer market as well as for military forces to protect against vector borne disease. Previous studies indicated that uniform efficacy could be compromised by strong permethrin IR caused by the two-IC haplotype, but the effect of lower levels of IR on uniform efficacy was unclear^16,36^. These *kdr* strains provided an opportunity to test a variety of resistance levels. We show that even the presence of one IC haplotype is enough to compromise the efficacy below the required minimum standard of 85%^37,38^. The loss of efficacy in the *kdr* strains varied and did not reach levels of the PR strain, which was confirmed here to result in complete efficacy loss. This is a critical issue that needs to be investigated further to define effective mitigations and conduct realistic risk assessment. We note that numerous mechanisms of repellency exist and the interplay between these mechanisms is complicated^52–54^. How these mechanisms play a role in what we observed, where even low levels of resistance result in efficacy failure, are unclear. We note that the spatial repellency of DEET and some volatile pyrethroids act over a distance to irritate, mask or confuse mosquitoes from contacting the human host but permethrin does not appear to act as a spatial repellent and instead requires contact with the material at which time mosquitoes choose not to bite^54,55^.

Overall, this portion of our study demonstrates that efficacy assessments of field performance are clearly overstated in uniform testing just as in field sprays when lab susceptible strains are assumed to be representative of even moderately resistant field strains. Resistant strains must also be used if real-world efficacy assessment is desired, as IR in *Ae. aegypti* is widespread worldwide.

This study also points to the importance of reporting the alleles present at both positions 1016 and 1534 together as a complete genotype rather than as separate loci for a population. Here we show that three *kdr* isolines with 100% 1534C each have significantly different resistance levels; VVCC has a low RR, VICC an RR ~ 20 and IICC an RR ~ 60. Thus, reporting the frequency of the 1534C loci alone indicates little about population level resistance. Reporting the 1016 and 1534 frequencies separately can also be misleading because the zero, one and two IC genotype groups have very different toxicological profiles and resistance levels. In the most extreme case, a population with 100% VI and 100% FC could be either 100% VIFC or 50% VVFF and 50% IICC. According to the different dose response curves and distinct wind tunnel performance we demonstrated, these two different *kdr* genotype populations would not have the same response to a given active ingredient that might be applied operationally and would be subject to different selective pressures in the field. It is reasonable to expect that the VIFC of the first population and the VVFF portion of the second population would be killed but that strong selection for the IICC portion of the second population would occur.

Heterologous expression in *Xenopus laevis* oocytes is the gold standard for isolating the effect of a mutation on a model sodium channel, but *VGSC* mutations in vivo exist in a much more complicated system^56,57^. Additional mutations of unknown effect, numerous expressed *VGSC* transcripts with different exon usage, unknown influence of the tipE protein and other mechanisms (like enzymatic or cuticular resistance) are present that can modulate activity and make direct correlation to phenotype difficult. Our limited transcript sequence analysis showed many additional *VGSC* mutations were present but presumably have little contribution to resistance as they do not appear consistently in IR phenotypes. The recently described V410L (or V419L) was found in all strains except the ORL and VVCC, including the least resistant isolines as well as the most resistant (including PR)^58,59^. It is increasingly found with the 1016I/1534C ensemble so presumably 410 L has physiological benefit, but its role is unclear. However, since the 410L mutation (and the 1534C mutation) was present in both susceptible and resistant strains, its use as a marker for strong pyrethroid resistance is not supported by this data. It is possible that mutations such as 410L (and/or 1532T) account for RR differences between the ORL baseline and the no-IC haplotype *kdr* strains. Notably the exons containing the 410, 1016 and 1534 mutation sites were present in all transcripts so we would expect the mutations to be fully expressed at the protein level regardless of the isoform.

It has been suggested that *kdr* assessment is a good predictor of resistance intensity and is beneficial due to ease of implementation and the ability to use dead specimens that cannot be used for bioassays^47^. Genotyping of *kdr* can certainly allow rapid screening of a wide area that would be difficult or even impossible to assess with bioassays and can rapidly identify areas with high frequencies of critical *kdr* genotypes for additional testing. Our previous area-wide study of Florida *Ae. aegypti* populations found a very strong correlation between the IICC frequency and phenotypic resistance level. Studies using disparate populations from other Western hemisphere locations have shown that *kdr* genotypes have distinct toxicologic responses^22,23,39^. This current study suggests that *kdr* genotype is a useful surrogate to estimate phenotypic resistance to pyrethroids in Western *Ae. aegypti*.

## Methods

### Mosquito strains

*Aedes aegypti* were collected as larvae from the area surrounding the St. Augustine Lighthouse (St. Augustine, Florida) in 2016 by Anastasia Mosquito Control District personnel as part of a state-wide collaborative *kdr* study^21^. The strain had the naturally occurring 410, 1016 and 1534 SNP mutations. Adults were allowed to freely mate and F1 generation eggs were collected using standard methods as described previously^21^. These F1 eggs were hatched and reared to the pupal stage where they were placed into individual emergence chambers consisting of 3.5 oz plastic cups (part #TK35, Dart Container Corp, Mason, WI), deionized H_2_O (30 mL) and a tulle mesh cover. Cups were maintained at 27 ^o^C until emergence, at which time the exuvia was removed by making a small slice in the tulle mesh and resealed with a 10% sucrose saturated cotton ball.

The exuviae were genotyped using the method described below to create six *kdr* isoline strains. Virgin, genotyped F1 males and females homozygous for zero, one or two *kdr* SNPs were grouped by genotype (1016VV/1534FF = VVFF, 1016VV/1534CC = VVCC, and 1016II/1534CC = IICC respectively) into cages, allowing free mating. These initial F1 isoline colonies were founded with at least 30 females each and were used to produce a larger F2 generation by another cycle of the same process to produce eggs of the VVFF, VVCC, and IICC genotypes. At this time, heterozygotes (VVFC, VIFC, VICC) were also produced by selectively breeding virgin genotyped males of one strain with virgin genotyped females of another strain. Organisms from generations F2-F5 were used for preliminary studies and to ensure VVFF, VVCC, and IICC were pure lines. At generation F5 the strains were mixed to ensure a uniform genetic background, allowed to mate and subsequently blooded to produce F6 eggs. Beginning at generation F6, pupae were placed into 10 mm test tubes containing 1.5 mL of deionized water (replacing the plastic cups), and F6 pupae were genotyped and separated into pure strains VVFF, VVCC and IICC. Subsequent generations of VVFF, VVCC and IICC were produced using the same individual emergence method before being allowed to freely mate. Every few generations, *kdr* screening of the VVFF, VVCC, and IICC strains was conducted to ensure purity. Eggs for the heterozygote lines VVFC, VIFC and VICC were produced using selective mating when needed as they cannot exist in continuous single-genotype colonies.

The CMAVE Orlando 1952 (ORL) susceptible strain and the CMAVE Puerto Rico (PR) pyrethroid resistant strain (equivalent to BEIResources #NR-48830) were maintained and used as control strains. Toxicology profiles and resistance levels for both laboratory strains have been determined previously^16,21,22,36^.

### Genotyping of pupal exuviae

Melt curve PCR assay (MCA) was used for genotyping the V1016I and F1534C mutations using previously described primers and methods^21,22,60,61^. Exuviae of each individually emerged adult was removed from the emergence chamber using a glass transfer pipette and placed in a 96-position deep well plate (Omni International, Kennesaw GA). Two glass homogenization beads (2.7 mm, Biospec Products, Bartlesville, OK) and 100 µL nuclease free water (NFW) was added to each well. Organisms with known *kdr* genotypes were used as controls and included the no *kdr* mutation ORL laboratory strain (genotype: VVFF, also 410VV), the resistant dilocus *kdr* PR strain (genotype IICC, also 410LL), an artificial heterozygote created from adding an ORL and PR strain mosquito into the same well (genotype: VIFC), and a NFW blank. Plates were homogenized for 60 s on a Bead Ruptor 96 homogenizer (Omni International, Kennesaw GA) and then spun for one minute at 805 × g. Separate MCAs for the V1016G and F1534C SNPs were assembled as described previously on an epMotion 5075 (Eppendorf, Hamburg Germany) with a final reaction volume of 10 µL (2 µL of template homogenate and 8 µL of SYBR-green, primer and NFW mastermix)^21,22^. Cycling and melt curve analysis were conducted on an Applied Biosystems StepOnePlus (96-well) or an Applied Biosystems QuantStudio 6 Flex (384-well) (Thermo Fisher Scientific, Waltham, MA) for 45 cycles. Genotype calls were made based on the presence of characteristic melting temperatures as previously described^21,60^.

### CDC bottle bioassay testing

Strains were reared to 3–7 days post-emergence and tested in the CDC bottle bioassay with two pyrethroids (permethrin (type I) and deltamethrin (type II)), and the organophosphate malathion. Doses per bottle were those recommended by CDC and the procedure followed the CDC guidance except as noted^9^. Three bottles of each diagnostic dose and three control (acetone only) bottles were used for each strain. In accordance with the guidelines, the same bottles were used for all strains to reduce variability due to differences in bottles and bottles were protected from light when not in use. Ten to twenty females were introduced into each bottle and mortality was recorded every five minutes for two hours or until all organisms in a bottle had died. Diagnostic doses and diagnostic times were those recommended by CDC: permethrin at 43 µg/bottle and 10 min, deltamethrin at 0.75 µg/bottle and 30 min and malathion at 400 µg/bottle and 15 min.

### Topical application assays

Application with permethrin, deltamethrin, and malathion was conducted on 3–7 days post-emergence females using methods described previously^15,21,22,46^. Initial assays were conducted with 10-fold gravimetrically prepared dilutions to determine the critical range (5–95% mortality) for each strain. Assays were then conducted on at least three different days for each strain and mortality was scored 24 h post-application. Analysis of this topical bioassay data was conducted in Prism v.9 using a 4-parameter logistic equation (GraphPad Software LLC, Boston, MA). Background mortality of 10% or less was subtracted using Abbott’s correction. The pesticide dose was converted to ng of active ingredient per mg of mosquito by dividing the applied dose by the average daily mass for the strain. Strains were considered significantly different if the 95% CIs around the LD_50_ did not overlap^62,63^. Resistance ratios were calculated by dividing the LD_50_ of the strain of interest by the LD_50_ of the ORL strain.

### Sample preparation for biochemical assays

Non-blood fed, adult female mosquitoes from the F7 generation were used for all biochemical assays. Groups of fifteen mosquitoes from each strain were weighed and homogenized with a disposable pestle in 100 mM KPO_4_ buffer (pH 7.2; 200 µL; Millipore Sigma, Burlington, MA). Homogenates were centrifuged at 18.4k × g for 10 min, and the supernatants were diluted with 160 µL of buffer. Specimens were maintained at 4 °C during processing and stored at −80 °C. Six groups were homogenized for each strain, and each group was assayed in triplicate. Flat bottom microplates were used for all assays. Each plate contained separate controls and calibrators as appropriate and were read on a Multiskan Sky Microplate Spectrophotometer (Thermo Scientific, Waltham, MA). Enzymatic activity was relativized by dividing the observed activity by the calculated protein concentration for each sample. Enzymatic assay data was analyzed by enzyme family in Prism using Brown-Forsyth and Welch ANOVA. Dunnett’s multiple comparison was used for means comparison to determine significant interactions.

### Total protein assay

Total protein of each mosquito group was assayed using the Pierce BCA kit (Thermo Scientific, Waltham, MA). The assay uses bicinchronic acid (BCA) for colorimetric protein detection at 562 nm. Samples were assayed using a 1:10 dilution following the manufacturer-provided procedures and analyzed with a bovine serum albumin standard curve with a maximum linear concentration of 2 mg/mL.

### Nonspecific esterase assay

The α- and β-esterase activity was measured using 1 (α)-napthyl acetate or 2 (β)-napthyl acetate solution respectively (Millipore Sigma, Burlington, MA). The naphthol produced by the cleavage of napthyl acetate produce chromogenic products in the presence of Fast Blue B. Fifty µl of 1 (α)-napthyl acetate or 2 (β)-napthyl acetate (56 g dissolved in 20 mL acetone and added to 80 mL KPO_4_ buffer) was added to 50 µL homogenate. All samples were diluted 1:10. Following a 10 min room temperature incubation, 50 µL of a Fast Blue/SDS solution (150 mg O-dianisidine tetraotized, 15 mL deionized H_2_O, 35 mL 5% SDS; Millipore Sigma, Burlington, MA) was added to each well, and the plate was read at 570 nm following a two minute incubation. Six-point standard curves ranging from 0 to 350 µM were constructed for both α-naphthol and β-naphthol.

### Oxidase assay

Oxidase activity was measured using a heme peroxidase assay with 3,3′, 5,5′ tetramethylbenzidiene (TMBZ) serving as the electron donor for the conversion of peroxides to water. One hundred µL of TMBZ was added to 50 µL of homogenate followed by 15 µL of 3% H_2_O_2_. Plates were incubated for 20 min and read at 620 nm. Cytochrome C (100 ng/µL; Millipore Sigma, Burlington MA) in 0.25 M sodium acetate buffer was used to generate a nine-point standard curve from 0 to 10 ng/µL.

### Glutathione-S-transferase assay

Glutathione-S-transferase activity was measured using a 1-chloro-2,4-dinitrobenzene (CDNB; Millipore Sigma, Burlington, MA) assay. One hundred µL of reduced glutathione (61 mg in 100 mM KPO_4_ buffer) was added to 100 µL of homogenate followed by 100 µL of CDNB (20 mg, 10 mL acetone, 90 mL KPO_4_). The plates were read once every minute for five minutes and monitored against the positive GST control (Cayman Chemical, Ann Arbor, MI) to ensure absorbance increased between 0.012–0.064/minute/well.

### Voltage gated sodium channel transcript sequencing

Nucleic acids were isolated as separate fractions from individual females of each strain using Quick-DNA/RNA Miniprep silica column kit (Zymo Research, Irvine, CA). Total RNA (11 µL) was reverse transcribed using the SuperScript IV First-Strand Synthesis System (Thermo Fisher Scientific, Waltham, MA) with random hexamer primers and an extended extension time (15 min) to allow for a long product. Polymerase chain reaction was performed using Q5 Master Mix (New England Biolabs, Ipswich, MA) and the primers CATTGTTGGCCATATAGACAATG and TCTGTCGTGCTTCTGAATCTG, which were designed to amplify the *Ae. aegypti VGSC* coding region. The cycling conditions were: 98 °C for 60 s, then 40 cycles of 98 °C for 10 s, 62 °C for 30 s, 72 °C for 6.5 min and then a final extension of 72 °C for 2 min. PCR products were examined for amplification of the full coding region (~ 6,500 bp) on a 4200 TapeStation System with Genomic DNA ScreenTapes (Agilent Technologies, Inc., Santa Clara, CA). Samples were cleaned using 100,000 Da MWCO centrifugal cellulose membranes (Millipore Sigma, Burlington, MA), with two washes of 400 µL of NFW, and then assessed for concentration on the TapeStation. Samples were subsequently barcoded, purified and pooled into groups using Nanopore sequencing kits NBD-104 and LSK-109 (Oxford Nanopore Technologies, Oxford, U.K.) per the manufacturer protocol. Between 135 and 158 ng of each purified and barcoded amplicon was included. Sequencing adapters were ligated to the barcoded samples and run for 48 h on a GridION sequencer using R9.4 flow cells (Oxford Nanopore Technologies, Oxford, U.K.). One thousand full length reads from each strain were aligned using Minimap2 or MAFFT to a predicted *VGSC* transcript from the *Ae. aegypti* genome using the coding region of XM_021852340.1:1717–8127 with the additional exon found in XM_021852342.1:148-181^64,65^. The alignment file was examined in IGV to assess the presence of SNPs at *VGSC* positions 410, 1016 and 1534^66^. Data for this analysis is found in the NCBI Sequence Read Archive under BioProject ID PRJNA832328.

### Aerial application of unsynergized pyrethrins

Field efficacy of a commercially available formulation of natural pyrethrins (Merus® 3.0, Clarke, St. Charles, IL), was tested by the Collier Mosquito Control District. Nine test stations, 25 feet apart, were placed in a 3 × 3 grid. Each test station was comprised of eight small field cages with ~ 20 1–5 day old female *Ae. aegypti* isoline, ORL or PR. Applications were performed by an MD 500 helicopter (MD Helicopters, Mesa, AZ) from a Micronair AU6539 atomizer (Sandown, Isle of Wight, U.K.) using the AGDisp modeling algorithm for Merus® 3.0 at the label recommended dose of 0.88 oz/acre. All equipment was current in calibration, and droplet size was measured to be within label range (D_v_ 0.5 = 38.99 μm; D_v_ 0.9 = 71.84 μm). To ensure coverage of the treatment zone, passes with a swath of 106 m (350 ft) were made perpendicular to the wind with application engaged from 50 m before through 50 m after the treatment zone. Initial knockdown was recorded at the test stations 10 min post-exposure, after which mosquitoes were brought into the laboratory and recovery was monitored every 15 min for two hours post-exposure, and additionally at eight, 24, and 48 h post-exposure.

### Wind tunnel assays

Formulated product testing was conducted at Anastasia Mosquito Control District in St. Augustine, FL using a fixed wind tunnel (Sigma Scientific, Alachua, FL) with filtration and variable speed control on F12-F16 generation mosquitoes. Tunnel air flow was maintained at a constant speed of 0.9 mph (0.4 m/s). Three formulations were tested: an unsynergized mix of pyrethrins (Merus® 3.0), a PBO synergized permethrin (Aqualuer® 20–20, Allpro Vector, St. Joseph, MO), and the organophosphate malathion (Fyfanon® EW, FMC Professional Solutions, Philadelphia, PA). The spray volume was applied through an air-shear nozzle capable of producing ULV spray (ADAPCO, Sanford, FL) at a constant pressure of 100 psi. This nozzle and pressure produce a D_v_ 0.5 of 17 µM. The formulated products were diluted in BVA oil to produce a spray flux through the 50.9 cm × 50.9 cm spray surface area equivalent to label rates of 0.88 (Merus® 3.0), 0.51 (Aqualuer® 20–20) and 2.1 (Fyfanon® EW) ounces per acre respectively when the volume (200 µL) was sprayed. One to two days before testing, each strain was knocked down with CO_2_, and 50 females were sorted into groups in paper tubes (6″ id × 1.5″). Open ends of these spray cages were covered in tulle mesh sealed with two tightly fitting 6.125″ id × 0.75 cm paper tubes. Mosquitoes were allowed to recover and given access to a 10% sucrose saturated cotton ball. Four cages of 50 females were tested with each formulation and strain. Four cages of each strain were also sprayed with just the diluent. On the day of testing, the wind tunnel and nozzle were thoroughly cleaned before placing two randomly selected cages into the tunnel on a test apparatus. Open cage ends were maintained perpendicular to the airflow by attachment to the test apparatus. Clean air (100 psi) was supplied to the nozzle, and the test dose was applied through the sample port of the nozzle. The nozzle flow was maintained for one minute after spray application. Cages were then removed from the tunnel and laid horizontally in 40 × 80 cm plastic pans (Del-Tec, Greenville, SC), and the spray time was noted for the pair of cages. Each cage was provided a 10% sucrose saturated cotton ball. Sprays continued in groups of two cages at a time until each had been sprayed. Knockdown (mosquitoes obviously dead or unable to right themselves) was recorded for each cage one hour post-spray and 24 h mortality was recorded using the same standard as for laboratory toxicology assays. Three replicates were performed for each treatment. Analysis of spray data was performed in Prism v.9 using means comparison.

### Uniform bite protection assay

A standardized human bite protection assay was conducted from March through August 2019 under an IRB approved human use protocol (University of Florida Institutional Review Board Protocol: 201700023) as described previously^36^. All methods were carried out in accordance with the IRB approved protocol, relevant guidelines, and regulations. This includes obtaining informed consent from all study participants. Three consenting volunteers with no known blood-borne disease or pregnancy participated in all exposures for all strains. Between 75 and 200 host-seeking female mosquitoes up to 10 days post-emergence were released into each of two cages and allowed to settle (15 min). Volunteers inserted each arm with a gloved hand into sleeves made of FRACU3, which was sealed at the overlap between glove and sleeve with tape; one sleeve was prepared with the standard dose of 0.52% permethrin, and the other was untreated. These sleeves were washed once before use under standard wash procedures^37^. The portion of the arms covered in sleeves were simultaneously placed into the cages to allow exposure. At the conclusion of the exposure (15 min), arms were removed and all the mosquitoes in the cage were aspirated into a collection chamber, anesthetized with CO_2_ and counted based on the presence or absence of a visible bloodmeal. To ensure that mosquitoes that had ingested partial bloodmeals were counted as having bitten through the material, all females without a visual bloodmeal were spread on white copy paper and then crushed beneath a second sheet of paper; any red blood spots were counted as individuals having bitten through. Bite-through fraction was calculated as the sum of blood fed (visible and by crushing) divided by the total number in the cage. Both susceptible ORL and resistant PR strains were included as known control strains. Data was analyzed by ordinary 2-way ANOVA (response variable: percent bite through; Factors: treated/untreated and strain) with means comparison as implemented by Prism v.9.

## Data Availability

Experimental data not included in the manuscript are available from USDA AgData Commons at: DOI 10.15482/USDA.ADC/25802143. Sequencing data is available from NCBI under BioProject ID PRJNA832328.
